# Melanization of *Dirofilaria immitis* Larvae in Different Culicid Species

**Published:** 2018-03-18

**Authors:** Gandílcia Aparecida de Carvalho, Rafael Antonio Nascimento Ramos, Rafael TrindadeMaia, Carlos Fernando Salgueirosa de Andrade, Candâmara Leucio Alves

**Affiliations:** 1 Academic Unit of Garanhuns, Federal Rural University of Pernambuco, Garanhuns, Brazil; 2 Department of Veterinary Medicine, Federal Rural University of Pernambuco, Recife, Brazil; 3 Department of Biological Sciences, Federal University of Campina Grande, Sumé, Brazil; 4 Department of Animal Biology, Biology Institute, State University of Campinas, Campinas, Brazil

**Keywords:** Mosquitoes, Immune response, Dirofilariasis, Vector

## Abstract

**Background:**

*Dirofilaria immitis* is an important filarioid transmitted by culicids. The vector role of these arthropods may be influenced by biological events as melanization against *D. immitis* larvae. This study aimed to detect the occurrence of melanization in *Culex quinquefasciatus*, *Aedes* (*Stegomyia*) *aegypti* and *Aedes* (*Stegomyia*) *albopictus* experimentally infected with *D. immitis*.

**Methods:**

Five populations (*Cx. quinquefasciatus* RECIFE (P1), *Ae. albopictus* RECIFE (P2), *Ae. aegypti* RECIFE (P3), *Ae. aegypti* CAMPINAS (P4) and *Ae. aegypti* ROCKEFELLER (P5)) were artificially fed with infected blood containing *D. immitis* microfilariae. Ten mosquitoes per day from each population were dissected for 14 days.

**Results:**

Melanized larvae of *D. immitis* were observed in all population except in P3. The period in which melanized larvae were found varied from the third to the 14^th^ day post-infection. Difference in the number of these larvae was observed between P5 and P2–P3 (P< 0.01), and between P5 and P4 (P< 0.05). Third-stage larvae of *D. immitis* were detected in all population 14 d post-infection, except in P3, which presented high mortality 24 h post-infection. The melanization observed in the populations herein studied did not indicate refractory since third-stage larvae were observed at the end of the experiment. Most likely, this immune response aimed to control the number of larvae, in order to maintain the equilibrium between vector-parasite.

**Conclusion:**

The melanization did not seem to be a limiting factor to the development of this filarioid in these local Brazilian Culicidae populations.

## Introduction

*Dirofilaria immitis* is an important nematode filarioid that causes a severe infection in dogs commonly known as heartworm disease. Although, other animal species as wild carnivores, cats and humans may be affected, dogs present a high epidemiological relevance (1). This filarioid is transmitted by culicid vectors, being species belonging to the genus *Culex*, *Aedes* and *Anopheles* the most important (2–5).

The vector role of Culicidae species may be influenced by biological events as immune responses against *D. immitis* microfilariae. Indeed, different types of immune response against parasites and pathogens affecting mosquitoes have been studied in some species (e.g., *Aedes aegypti*, *Ochlerotatus trivittatus*, *Anopheles quadrimaculatus*, and *Armigeres subalbatus*) (6–10). For example, the melanization is an important immune event that occurs inside the mosquito vector. This phenomenon is characterized by an initial reaction involving the lysis of hemocytes close the surface of the parasite prior to the deposition of pigments (8, 11). The melanization may impair the flow of nutrients that are essential to the microfilariae survival and development. Moreover, it may difficult the release of toxic oxygen metabolites causing the death of the parasite (12).

The melanization of *D. immitis* larvae was reported in *A. subalbatus*, *Ae. aegypti* and *Cx. quinquefasciatus* (11, 13, 14). This immune response may reduce the vector ability of some populations of mosquitoes in transmit parasites, including *D. immitis* (15). On the other hand, species belonging to the genus *Aedes* use this phenomenon to restrict the larval development, enhancing its potential as vector (16, 17). A melanization is an event that may vary depending on the population studied, and in Brazil, this phenomenon has been poorly studied in the local Culicidae population.

Therefore, the aim of this study was to assess the occurrence of melanization in different populations of culicids (*Cx. quinquefasciatus*, *Ae. albopictus* and *Ae. aegypti*) experimentally infected with *D. immitis* microfilariae to evaluate the potential role of these species as vectors. In addition, biological implications of these findings have been discussed.

## Materials and Methods

### *Dirofilaria immitis* microfilariae

Infected blood containing *D. immitis* microfilariae was obtained from a positive dog diagnosed at microscopic and molecular analyses. The animal was a three-yr-old male that lived in the metropolitan region of Recife (7°45′0“S and 34°51′0”W), state of the Pernambuco, Brazil.

### Mosquito populations and experimental infection

Five populations belonging to three different culicid species were used in this study: *Cx. quinquefasciatus* RECIFE (P1), *Ae. albopictus* RECIFE (P2), *Ae. aegypti* RECIFE (P3), *Ae. aegypti* CAMPINAS (P4) and *Ae. aegypti* ROCKEFELLER (P5).

Overall, 6000 female mosquitoes (1200 per each population, test group =900 and control group = 300) aged from three to seven days were used (18). The artificial blood meal was performed as previously described (4, 5). Briefly, mosquitoes were fed for two hours with infected blood containing about 2000 microfilariae/ml of *D. immitis*. After blood meal, mosquitoes were maintained under controlled conditions of temperature (28±2 °C) and relative humidity (>70%).

### Mosquito dissection and microscopic examination

Ten mosquitoes per day from each population test were dissected during 14 days. Daily, dead mosquitoes were discarded and at the end of the experiment the remaining ones were dissected. Briefly, specimens were fixed on slides containing a drop of 0.9% physiological saline solution, dissected with a sterile scalpel and immediately examined under a light microscope (Olympus BX41 TF) at different magnifications. All stages of *D. immitis* larvae were morphologically identified (19) and the presence of melanized larvae was recorded.

### Data analysis

The percentage of infected mosquitoes, as well as number of melanized larvae, was calculated. The statistical analysis was performed through the Partitioning Qui-square test using the software BioEstat 2.0 (20).

## Results

The overall results with the number of infected females and melanized larvae are shown in Table 1. Melanized larvae were detected in all population except in P3 and the highest number was detected in P5 followed by P1, P4, and P2, respectively. In general, the period in which these melanized larvae were found varied from the third to the 14 ^th^ day post-infection. The developing stage in which they were observed is reported in Table 2. Difference in the number of melanized larvae was observed between P5 and P2 (P< 0.01), and between P5 and P4 (P< 0.05).

**Table 1.** Infected females with *Dirofilaria immitis* microfilariae, and presence of melanized larvae

	**P1**	**P2**	**P3**	**P4**	**P5**
Females with *D. immitis* microfilariae (%)^*^	13.47 (111/824)	15.63 (131/838)	17.55 (149/849)	11.57 (95/821)	16.97 (149/878)
**Females with *D. immitis* melanized larvae** ^**^ **(%)**	10.81 ^a^ (12/111)	0.76 ^b^ (01/131)	0 ^b^ (0/149)	5.26 ^a, c^ (05/95)	19.46 ^a, d^ (29/149)

(*Number of females infected/Number of females dissected,

** Number of females with melanized larvae/Number of females infected. Different letters indicate statistically significant difference)

**Table 2.** Developing stage of the melanized larvae in different populations analyzed

	**First stage**	**Salsichoid and second stage % (n/N)**	**Third stage**
**P1**	-	100 (12/12)	-
**P2**	-	100 (1/1)	-
**P4**	80 (4/5)	-	20 (1/5)
**P5**	68.9 (20/29)	31.1 (9/29)	-

Interestingly, third-stage larvae of *D. immitis* were detected in all population 14d post-infection, except in P3, which presented a high mortality (70.7%) 24 h post-infection. The P2 presented the first L3 nine days post-infection, whereas for P4 and P1 third-stage larvae were observed ten and 12d post-infection, respectively. Finally, for P5 *D. immitis* L3 were retrieved only 14d post-infection, but melanized first-stage larvae were detected until the end of the experiment.

The infected mosquitoes showed total or partial destruction of the cells of Malpighian tubules (MT) in the parasitized areas. The membrane of the cells presented damaged, most likely due to the migration of the larvae from the tubules to the head and proboscides. During this migration, the reaction of melanization was not observed.

During the whole study, the mortality of mosquitoes of control groups was approximately 3% for all groups herein analyzed.

## Discussion

In this study, the melanization of *D. immitis* larvae in different culicid populations and species was studied. The melanization that occurs in *D. immitis* larvae in some mosquitoes suggests that these specimens may be resistant to the parasitism by this filarioid, depending on the percentage of melanized larvae (16). Indeed, in populations such as *Ae. aegypti* ROCKEFELLER this phenomenon is more evident (21, 22).

In general, the development of *D. immitis* larvae in culicids occurs in 14d, from microfilariae to infective-stage larva. However, depending on the mosquito population, several biological events (e.g., blood coagulation, microfilaria capture and melanization) may occur to control the number of larvae in development inside the Malpighian tubules (5–16). These mechanisms are important to regulate the number of infected mosquitos, which will influence in their survival and vectorial competence (23).

In the present study, almost all populations (except P3) presented melanized larvae, but in all of them (except P3) third-stage larvae of *D. immitis* were detected at the end of the experiment. Most likely, in this population microfilariae were destroyed at the first 24 h post-infection, therefore, no melanized larvae and infective-stage larvae were observed. This is an important finding, especially because all populations and species herein studied are susceptible and allows the development of *D. immitis* microfilariae; accordingly, these populations are considered potential vectors of *D. immitis*.

The susceptibility of *Ae. aegypti* species for *D. immitis* infection is a characteristic controlled by the f ^1^ gene (24). Females that present the patterns FiFi or Fifi are refractory to the infection. Indeed, these genes may influence the physiology of Malpighian tubules, and consequently the development of the parasite. Most likely, this genetic factor has an important role in the phenomenon of melanization, affecting the vector competence of culicids (8, 25). In fact, genetic differences may be observed in individuals of the same species (eg: *Ae. aegypti*) and different populations (24). These differences may be the cause of the findings of melanization herein reported for the same culicid species (ie, P4 and P5). This variation within a mosquito species contributes to enhancing the vector role of some populations from different geographical areas (26).

The structural alterations herein observed in infected mosquitoes may play an important role in the development of *D. immitis* larvae. The mortality of infected mosquitoes probably may occur due to two reasons: i) the invasion of the MT cells by the microfilariae and ii) the escape of the infective larvae from the MT and their movement to the head and mouthparts (27). Therefore, when the parasite load in the MT is higher, an excessive mortality (almost 100%) may occur (28, 29).

In this study, the parasitism by *D. immitis* microfilariae had a great impact on females of P3, influencing the survival of specimens which dead (almost 70.7%) 24h post-infection.

## Conclusion

The melanization observed in the populations herein studied did not indicate refractory, since third-stage larvae were detected at the end of the experiment. Most likely, this immune response herein observed aimed to control the number of larvae in development, which is important to maintain the equilibrium between vector-parasite enhancing the potential of these specimens as vectors. All population herein studied, except P3, may act as a potential vector of *D. immitis* in Brazil. The authors declare that there is no conflict of interest.
